# High-Dietary Fiber Diet Reduces Arsenic Oral Bioavailability and Health Risk from Soils by Regulating Gut Microbiota and Intestinal Barrier Function

**DOI:** 10.3390/foods15111961

**Published:** 2026-06-02

**Authors:** Shuo Chen, Lei Han, Enfeng Liu, Hongbo Li, Jie Li

**Affiliations:** 1 Shandong Province Key Laboratory of Emerging Contaminants Risk Prevention and Control, College of Geography and Environment, Shandong Normal University, Jinan 250358, China; 2 Shandong Provincial Geo-Mineral Engineering Exploration Institute, Shandong Provincial Bureau of Geology & Mineral Resources, Jinan 250014, China; 3 State Key Laboratory of Pollution Control and Resource Reuse, School of Environment, Nanjing University, Nanjing 210023, China; 4 Jiangsu Key Laboratory of Vehicle Emissions Control, School of Environment, Nanjing University, Nanjing 210023, China

**Keywords:** arsenic, bioavailability, intestinal barrier, gut microbiota, high-dietary fiber diet

## Abstract

Unlike conventional energy-intensive physical/chemical soil remediation, dietary regulation of As oral bioavailability represents a cost-effective, sustainable downstream intervention in environmental risk management and control. However, how distinct dietary structures regulate As bioavailability remains unelucidated, hindering a holistic understanding of corresponding exposure and health risks. To address this, a mouse bioassay was conducted to evaluate the relative bioavailability (RBA) of As in two soils with four typical diet structures (high-fat, high-protein, high-carbohydrate, and high-dietary fiber diets). The results showed that although the four diets promoted the gastrointestinal As dissolution by 1.1–1.7-fold, the high-dietary fiber diet decreased As-RBA by 9.49–13.2% and lowered the health risk by 0.50–0.70-fold, which was more effective than high-protein and high-carbohydrate diets. The decrease was associated with lower intestinal permeability, which correlated with a significant increase in the relative abundance of *Roseburia* and *Lachnospiraceae*, and a decrease in the apoptosis rate of mouse intestinal epithelial cells. In contrast, a high-fat diet increased As-RBA by 8.72–11.9% and raised the health risk by 1.33–1.38-fold, which was associated with a significant proliferation of *Dubosiella* and a significant inhibition of *Roseburia*. This study shows that a high-dietary fiber diet is associated with reduced As exposure and potential health risks, in parallel with favorable changes in gut microbiota, oxidative status, and intestinal permeability.

## 1. Introduction

As a class I carcinogen, arsenic (As) has attracted worldwide health attention [1]. Humans can be exposed to arsenic through a variety of ways, including drinking water, food intake, and inadvertent soil intake [2]. Long-term exposure to As may cause a series of diseases such as cardiovascular diseases, diabetes, and cancers [3,4]. Among pathways, incidental ingestion of contaminated soil is an important source for child exposure to As via frequent hand-to-mouth behavior [5]. Soil As pollution has become a tough challenge for environmental management in many countries worldwide. The traditional repair technology is expensive and difficult to implement on a large scale, so reducing the risk from the source of exposure has become a feasible supplementary solution.

To accurately assess and regulate the health risks of As exposure, it is necessary to consider the As content absorbed into the circulation by the gastrointestinal tract in soils. The bioaccessibility of As refers to the portion dissolved in the gastrointestinal fluid, while the bioavailability refers to the portion absorbed into the systemic circulation. Among them, bioaccessibility does not always predict bioavailability, because intestinal barrier function and intestinal microbiota regulate absorption efficiency. Accidental ingestion of As-contaminated soils usually co-exists with food in the gastrointestinal tract, and some food components have been reported to affect As absorption, which in turn affects As bioavailability. For example, FeCl3 supplementation can significantly lower 5.0–50.0% of As relative bioavailability (RBA) in soil, as it promotes the formation of As-Fe complexes to block As from crossing the intestinal barrier [6]. The presence of Ca aspartate reduced 41–72% of As-RBA in indoor dust by inhibiting the expression of phosphorus transporters in the duodenum of mice [7]. Folic acid supplementation can promote As methylation and increase the content of dimethyl arsenic acid in urine [8]. Notably, 5% fructooligosaccharides supplementation significantly increased the As concentration in the liver [9]. Most current studies focus on nutrients such as mineral elements and bioactive components with lower daily intake, and the effect of the main food components (such as protein, fat, and carbohydrates) on As oral bioavailability remains unclear.

Protein, fat, and carbohydrates have important impacts on children’s growth and development [10]. Recently, the diet for children has gradually tended to be high-fat in many countries, such as China and the USA [11,12,13]. Therefore, the impact of different dietary patterns on As-RBA deserves attention. Food digestion in the gastrointestinal tract involves a series of physical and chemical effects and subsequent intestinal absorption of As. Studies have shown that compared with the normal diet, a high-fat diet can significantly reduce the As content in the feces of mice [14]. Previous studies have also reported contradictory effects of dietary components on As bioavailability and bioaccessibility. Protein supplementation increased the As bioaccessibility by 15.8–35.4% via in vitro experiments [15]. However, Gruber et al. (2012) found that protein intake can promote the excretion of As in the human body [16]. For people exposed to As, food affects the absorption and accumulation of As by regulating the metabolism of As. Although As contamination in the environment cannot be eliminated through dietary regulation, it is a low-cost, sustainable, and complementary strategy that helps reduce As oral bioavailability and mitigate health risks at the individual level. However, the mechanism of the effect of nutrients on As-RBA in soil is still unclear. In addition, due to physical health considerations, children’s dietary fiber intake needs to be taken seriously [17,18]. Till now, the effect of dietary fiber intake on As absorption remains unknown. To effectively regulate the health risks caused by As exposure, it is necessary to clarify how food components affect As-RBA.

The intestine serves as a pivotal absorptive organ, and the integrity of the intestinal barrier is closely associated with As oral bioavailability [19]. For example, Zhang et al. (2023) found that *Akkermansia* enhanced intestinal barrier function by promoting the increase in cecal goblet cells and mucus secretion, resulting in a decrease in As bioavailability [9]. The composition of the intestinal microbiota tends to shift in association with food. When the energy provided by dietary protein was increased to 52%, the abundance of Bifidobacterium, Bacteroides, Parabacteroides, and Oscillospira in the intestinal tract of mice increased, showing increased intestinal permeability and systemic inflammation [20]. Rats fed with a high-fat diet had a lower total bacterial density in the intestines, and the relative proportion of Bacteroidales and Clostridiales increased, accompanied by the development of colonic inflammation [21]. In addition, the intake of high-fat and high-protein diets can lead to oxidative damage [22,23,24]. Intestinal flora imbalance, inflammation, and oxidative damage can destroy the integrity of the intestinal barrier, thereby increasing intestinal permeability and promoting the absorption of metal elements [25]. Therefore, we hypothesized that dietary components are associated with variations in regulating intestinal barrier function by altering the composition of gut microbiota, thereby affecting intestinal absorption of As.

These data indicate the need for dietary components, which can confirm their role in human health risk assessment from exposure to As-contaminated soils. Therefore, the aims of this study were to investigate the effects of dietary components on the intestinal absorption of As in contaminated soil by mouse bioassay and to elucidate its main mechanisms. We hypothesize that the association between diet and As bioavailability is mediated by changes in gut microbiota and intestinal barrier function. The specific research objectives include the following: (1) to evaluate the effects of high-fat, high-protein, high-carbohydrate, and high-dietary fiber diets on As-RBA in mice exposed to As-contaminated soil; importantly, (2) to explore potential associations between gut microbiota composition, intestinal barrier function, and As bioavailability under different dietary patterns to provide preliminary insights into the underlying mechanisms. The purpose of this study was to elucidate how major dietary patterns regulate As oral bioavailability and to explore the effects of As on intestinal microbiota and intestinal barrier function. The results of this study will provide a mechanistic insight into the interaction between diet and As, which provides a way to reduce the risk of human exposure to As by protecting the stability of the intestinal barrier.

## 2. Materials and Methods

### 2.1. Soil Samples

Two soil samples were used in this study, with soil A collected from a mining-contaminated area in Yunnan Province and soil B sampled from an agricultural area affected by metallurgical activities in Henan Province, China. The collected soil samples were naturally air-dried, ground, and sieved using a 250 μm nylon mesh. Soil samples (~0.5000 g) were digested in triplicate using the US EPA 3050B method [9]. Total As, Fe, Ca, and Mn content in digested samples was determined using inductively coupled plasma mass spectrometry (ICP-MS, iCAP RQ, Thermo Scientific, Madison, WI, USA) and inductively coupled plasma optical emission spectrometry (ICP-OES, Optima 5300DV, PerkinElmer, Waltham, MA, USA). The As concentration in soil A and soil B were 381 ± 7.71 mg kg^−1^ and 121 ± 9.58 mg kg^−1^, respectively (Supplementary Materials, Table S1). Details on the physicochemical properties of the soils were provided in Table S1. For quality assurance and quality control (QA/QC), a standard reference material (SRM NIST 2711a, National Institute of Standards and Technology) was included, with measured As concentration being 92.1 ± 0.86 mg kg^−1^ (*n* = 3), which was similar to 86.0 ± 0.8 mg kg^−1^ reported by a previous study, Li et al. (2016) [4].

### 2.2. Mouse Assay

#### 2.2.1. Mouse Chow Preparation

To reduce the intervention of mixed dietary variables, a simplified single-component high-concentration diet was used to modify the dietary composition of mice. With reference to the high dose levels commonly found in previous reports, rice, wheat fiber, lard, and protein powder were mixed with mouse chow (catalog number: F010201) to determine the effects of high-carbohydrate (74.9% energy from carbohydrate, 74.9%E), high-dietary fiber (9.75%E), high-fat (40.0%E), and high-protein (42.2%E) diets on As bioavailability [26,27]. The nutritional structure of the different diets is shown in Table S2. Specifically, the diets were prepared by combining food and mouse chow in specific ratios (Table S3). After each food was thoroughly mixed with mouse chow separately, 12 g of As-contaminated soil was accurately weighed and added to 348 g of the revised mouse chow mixture and again thoroughly mixed. Subsequently, the mixture was made into pellet form using Milli-Q water (Millipore, Burlington, MA, USA) and processed by freeze-drying to obtain the mixed diets (~4 g dry weight). Mouse chow was noted as the no-As-exposure group, and the As-contaminated soil amended diet was marked as the basal diet group. Also, sodium arsenate was added to mouse chow at 2–50 mg As kg^−1^, serving as a reference for soil As-RBA calculation. A linear dose–response curve (DRC) for As accumulation in the liver and kidneys was established for As doses using sodium arsenate (Figure S1), which indicates the suitability of selecting this endpoint for As-RBA measurements.

Background As levels in high-protein, high-fat, high-carbohydrate, and high-dietary fiber diets (without the addition of As-contaminated soil) were 0.01 ± 0.00 mg kg^−1^, 0.17 ± 0.14 mg kg^−1^, 0.08 ± 0.01 mg kg^−1^, and 0.00 ± 0.01 mg kg^−1^, respectively. As concentrations in food were>3 orders of magnitude lower than those in soils and interfered less with the soil As-RBA determination.

#### 2.2.2. As Concentration Analysis

Soil As-RBA administered with different food components (high-protein, high-fat, high-carbohydrate, and high-dietary fiber diet) was determined using a steady-state dosing approach via diet over a 14-day period. Female mice (Balb/c, 18–20 g body weight, bw) were purchased (Shandong Pengyue Experimental Animal Technology Co., Ltd., Jinan, China) and acclimated (12/12 h light/dark cycle, 25 °C and 50% humidity) for 1 week with Milli-Q water and uncontaminated mouse chow supplied ad libitum. During acclimation, all mice were cared for according to the Ethical Review Form for Laboratory Animals of Shandong Normal University and approved by the Ethics Committee for Animal Experiments of Shandong Normal University under approval NO. AEECSDNU2024008.

After acclimation, mice were fasted overnight and randomly separated into individual plastic cages with six individual mice in each group for treatment. At 9:00 am each day, ~4.00 g of the above different diets was provided to each mouse for free consumption. After a 14-day dietary exposure period, the mice were fasted overnight again, the final body weights were recorded and compared with the initial body weights, and the diet consumption per day was recorded (Figure S2). Subsequently, mice were sacrificed via blood collection under isoflurane anesthesia (5%), and samples of liver, kidneys, ileum, jejunum, and cecum contents were collected. Liver and kidneys were freeze-dried and digested according to US EPA 3050B method and then measured for As by ICP-MS.

The As-RBA was calculated as follows:

As-RBA= (Liver and kidneys As_Soil_ × As dose_sodium arsenate_) × 100%/(As dose_Soil_ × Liver and kidneys As_sodium arsenate_), (1)

where liver and kidneys As_Soil_ and liver and kidneys As_sodium arsenate_ are As concentrations in liver and kidneys (mg kg^−1^) after 14 d doses of soil and sodium arsenate to mice, respectively, and As dose sodium arsenate and As dose Soil are the As doses of sodium arsenate and soil exposure (mg As kg^−1^ bw), respectively. For QA/QC, SRM NIST 2710a was added into the basal diet to achieve an As concentration of 5 mg As kg^−1^, with the As-RBA being 37.1 ± 6.14%, consistent with 33.2% reported by Li et al. (2021) [28].

### 2.3. Arsenic Solubility Assessment

To further evaluate As-RBA differences across dietary components, soil As solubility in simulated gastrointestinal fluids in the presence of dietary components was assessed using the physiologically based extraction test (PBET) method [29,30,31]. Relevant experimental details were provided in Text S1 of the Supplementary Materials. Then, a zeta potential analyzer (Zetasizer Nano ZSE, Malvern Instruments Ltd., Malvern, Worcestershire, UK) was used to measure the zeta potential of the simulated intestinal fluid. The higher absolute zeta potential value indicates that the repulsion between particles is greater and the particulate matter is more stable [32].

### 2.4. Characterizing Gut Barrier

The gut is the most important absorptive organ of mammals, and the integrity of the intestinal barrier is closely correlated with its absorptive capacity [9]. The effect of dietary structure on the intestinal barrier was determined by staining paraffin-fixed ileum tissue sections with terminal deoxynucleotidyl transferase dUTP nick labeling (TUNEL). Under the fluorescence microscope, the nuclei of apoptotic cells appear red, while intact cells appear blue. ImageJ software (version 1.53e) was used for image processing and cell analysis. Detailed procedures are provided in Text S2.

Diamine oxide (DAO) was adopted as a biomarker reflective of intestinal permeability [33]. Ater centrifuged at 2000 rpm for 10 min at 4 °C, the upper serum layer of fresh blood samples was assayed for DAO enzyme activity, which was measured using a diamine oxidase kit (A088-1-1, Nanjing Jiancheng Institute of Biotechnology, Nanjing, China).

### 2.5. Oxidative Injury

The intake of metal(loid) with high levels of dietary components usually produces oxidative stress damage to the organism, leading to changes in intestinal permeability [34,35,36]. This study determined superoxide dismutase (SOD) and acetylcholinesterase (AchE) via jejunum samples to assess oxidative damage in the mouse intestine. An increase in enzyme activity indicates damage by reactive oxygen species, while a decrease in enzyme activity represents oxidative damage beyond the limits of the enzyme activity system [35,37]. Details of the enzyme activity assay are provided in Text S3.

### 2.6. Characterization of the Gut Microbial Community

Differences in dietary composition can lead to alterations in metal(loid) bioavailability by interfering with changes in the gut microbial community. This effect was, therefore, described by characterizing the structure of the gut microbial community in mice with different dietary exposures. DNA was extracted from mouse cecum samples using a faucal genomic DNA kit (CW2092S, CoWin Biotech, Beijing, China), and the extracted DNA was amplified by PCR (GeneAmp9700, ABI, Foster, CA, USA) to the V3-V4 region of bacterial 16s rRNA. Primers 338F (5′-ACT CCT ACG GGA GGC AGC A-3′) and 806R (5‘-GGA CTA CHV GGG TWT CTA AT-3′) were selected to ensure broad coverage and specificity for a wide range of bacterial taxa. PCR amplification products were analyzed by 2% agarose gel electrophoresis, cut and recovered using the AxyPrep DNA Gel Recovery Kit (AP-GX-50, Corning, NY, USA), and finally quantified using the QuantiFluorTM-ST Blue Fluorescence Quantification System (Promega, Madison, WI, USA). The amplified products were sequenced using the Illumina Nextseq 2000 platform from Majorbio, Shanghai, China.

### 2.7. Data Processing

All data were shown as mean ± standard deviation. Each digestion batch included at least three blanks, and each sample was measured three times during the analysis using ICP-MS, with a relative standard deviation of <0.5%. Recoveries ranged from 96.1 to 102.8% for every 20 samples measured using ICP-MS for standard solutions. A Tukey HSD test was used for one-way analysis of variance (ANOVA), and significant differences at α = 0.05 were determined using IBM SPSS software (version 26.0, Chicago, IL, USA). Normality and homogeneity of variance were ensured by the Shapiro–Wilk test and Levene’s test, and no data points were categorized as outliers.

## 3. Results and Discussion

### 3.1. Effect of Diet on As Accumulation in Mouse Tissue

**Figure 1:**
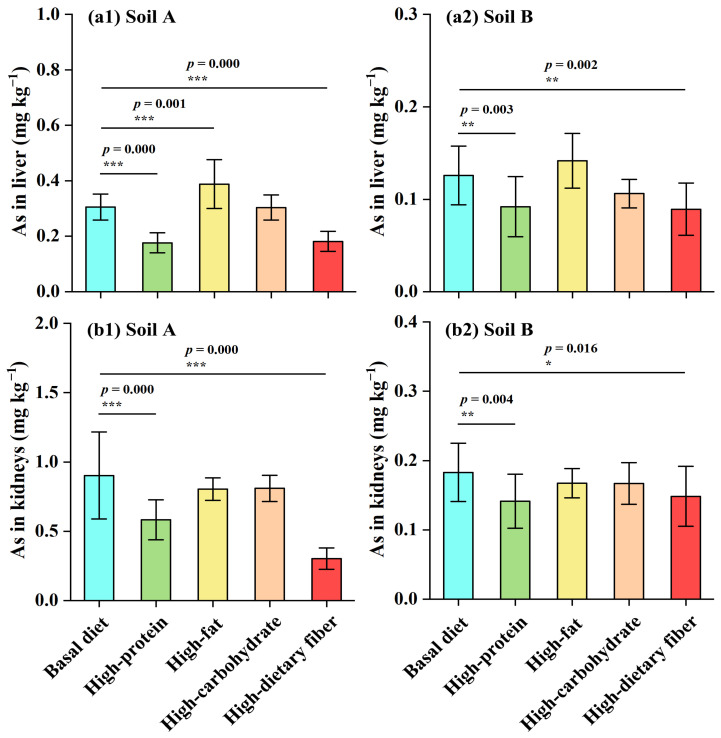
Effects of different diets on As concentrations in liver (**a1**,**a2**) and kidneys (**b1**,**b2**). Each bar represents the mean ± standard deviation of six replications. Basal diet: mice fed diet amended with As-containment soil. High-protein: mice fed diet amended with both As and 42.2%E protein. High-fat: mice fed amended with both As and 40.0%E fat. High-carbohydrate: mice fed amended with both As and 74.9%E carbohydrate. High-dietary fiber: mice fed amended with both As and 9.75% dietary fiber. Marks above the bars indicate significant differences at *p* < 0.05 (*), *p* < 0.01 (**), and *p* < 0.001 (***), respectively.

With the no-As-exposure group, As concentration in the liver (0.07 ± 0.01 mg kg^−1^, *n* = 6) and kidneys (0.11 ± 0.04 mg kg^−1^, *n* = 6) were low. Compared with the basal diet group (0.31 ± 0.03 mg kg^−1^ and 0.13 ± 0.02 mg kg^−1^ in soil A and soil B, respectively), high-protein (0.18 ± 0.02 mg kg^−1^ and 0.09 ± 0.02 mg kg^−1^) and dietary fiber (0.18 ± 0.02 mg kg^−1^ and 0.09 ± 0.02 mg kg^−1^) significantly reduced As concentration in the liver (*p* < 0.05) (Figure 1). In contrast, the high-fat diet increased As in the liver of mice exposed to soil A (0.39 ± 0.06 mg kg^−1^, *p* < 0.05) and soil B (0.14 ± 0.02 mg kg^−1^). In the kidneys, the basal diet group had the highest As concentration (0.90 ± 0.21 mg kg^−1^ and 0.18 ± 0.03 mg kg^−1^ in soil A and soil B, respectively), while high-protein (0.58 ± 0.10 mg kg^−1^ and 0.14 ± 0.03 mg kg^−1^, *p* < 0.05), high-fat (0.80 ± 0.05 mg kg^−1^ and 0.17 ± 0.01 mg kg^−1^), high-carbohydrate (0.81 ± 0.06 mg kg^−1^ and 0.17 ± 0.02 mg kg^−1^), and high-diet fiber (0.30 ± 0.05 mg kg^−1^ and 0.15 ± 0.03 mg kg^−1^, *p* < 0.05) reduced As concentration in the kidneys. Although the accumulation of As in the liver showed some differences between soils due to differences in soil types, increasing the intake of protein and dietary fiber may be an effective way to reduce As accumulation in tissues after soil As exposure.

### 3.2. Effect of Diet on As-RBA and As Bioaccessibility

**Figure 2:**
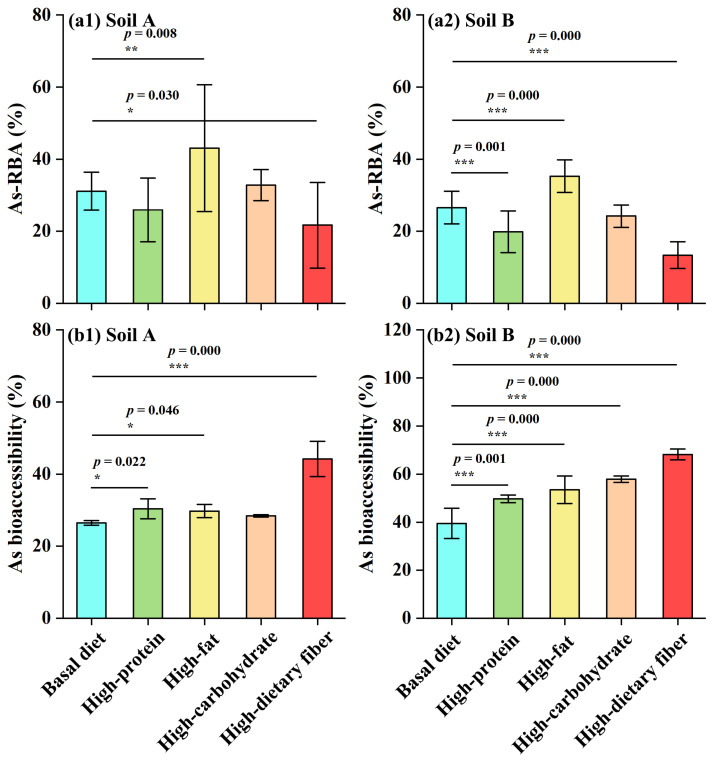
Differences in relative bioavailability and As bioaccessibility in intestinal phase under different diets. (**a1**,**a2**) represent the effect of dietary structure on As-RBA in soil A and soil B, respectively. (**b1**,**b2**) represent the effect of diet structure on bioaccessibility in intestinal phase in soil A and soil B, respectively. Marks above the bars indicate significant differences at *p* < 0.05 (*), *p* < 0.01 (**), and *p* < 0.001 (***), respectively.

Based on the As accumulation in liver and kidneys, the As -RBA of mice exposed to As-contaminated soil for 14 days under different dietary conditions was quantified (Figure 2(a1,a2)). Compared to the basal diet (31.1 ± 3.52% and 26.5 ± 3.04% in soil A and B, respectively), As-RBA was significantly higher for the high-fat diet (43.1 ± 11.7% and 35.3 ± 3.00%, *p* < 0.05), while significantly lower for high-dietary fiber (21.7 ± 7.95% and 13.3 ± 2.48%, *p* < 0.05). Meanwhile, a high-protein diet decreased As-RBA in soil B (19.8 ± 3.85%, *p* < 0.05).

Orally ingested As is released from the soil in the gastrointestinal tract and then absorbed for systemic distribution and metabolism [38]. To investigate the intestinal solubility of As under the influence of different dietary components, As bioaccessibility was obtained by the PBET method (Figure 2(b1,b2)). For the basal diet group, As bioaccessibility was 26.5 ± 0.42% in soil A and 39.5 ± 4.19% in soil B, respectively. Notably, high-protein (30.4 ± 1.83% and 49.7 ± 1.07%), high-fat (29.8 ± 1.23% and 53.5 ± 3.79%), high-carbohydrate (28.4 ± 0.21% and 57.9 ± 0.89%), and high-dietary fiber diet (44.2 ± 3.25% and 68.2 ± 1.53%) significantly increased the As bioaccessibility (*p* < 0.05). A high-dietary fiber diet demonstrated the highest As bioaccessibility. This is closely associated with the cereal dietary fiber’s well-defined interfaces, which substantially augment the availability of adsorption sites [18]. Furthermore, fatty acids hydrolyzed from dietary fat and bile are capable of forming micelles in the intestine [32]. After adsorbing the dissolved As in the intestinal phase, these micelles or particulate matter can be stably suspended by virtue of the electrostatic repulsion [39].

To explore associations between food components and particulate matter formation, the zeta potential of intestinal extract was determined (Figure S3). When dietary components were adjusted in the PBET, the absolute zeta potential value of high-fat (−26.2 ± 0.89 mV and −33.4 ± 0.10 mV in soil A and B, respectively), high-protein (−25.1 ± 0.69 mV and −22.1 ± 0.38 mV), high-carbohydrate (−23.1 ± 1.19 mV and −21.6 ± 0.46 mV), and high-dietary fiber diet (−18.2 ± 0.51 mV and −22.9 ± 0.72 mV) were significantly higher than those of the basal diet group (−2.26 ± 0.91 mV and −10.2 ± 1.05 mV, *p* < 0.05). The supplementation of dietary components promoted the formation and stabilization of particulate matter in the intestinal phase. However, As-RBA and As bioaccessibility of all treatment groups showed significant differences (Figure S4). This is because the increase in As bioaccessibility only indicates an increase in As solubility in gastrointestinal fluid, while As-RBA depends on intestinal absorption and barrier integrity. These results showed that, although the high-protein and high-dietary fiber groups promoted the dissolution of As in the gastrointestinal tract, they inhibited the absorption of As in the intestine through some special mechanisms and were associated with reduced As-RBA.

### 3.3. Effect of Diet on Gut Microbiota

Gut microbiota serves as a barrier in limiting the absorption of heavy metals [40,41]. However, the dietary modifications can alter the gut microbial community and disrupt the gut permeability [20,21,42,43]. Alpha diversity analysis revealed significant differences between the high-fat diet group and the basal diet group (*p* < 0.05, Figure S5). Beta diversity exhibited clear separation among the four dietary treatment groups (Figure S6). Notably, the gut microbial community structure of the high-fiber diet group was similar to that of the basal diet group and the arsenic-free exposure group. Different dietary patterns exerted significantly distinct effects on the stability of gut microbial community structure. Given the importance of the ratio of Firmicutes to Bacteroidota (F/B) in maintaining normal gut function, F/B was selected to represent the change in phylum-level microbial community structure [44]. Compared with the basal diet group (3.30 ± 0.70), the F/B value was significantly increased in the high-protein (22.6 ± 10.4) and high-fat (21.9 ± 4.30) diet groups (*p* < 0.05) (Figure S7). This increase in F/B value is associated with intestinal microbial imbalance and the occurrence of intestinal diseases [45].

**Figure 3:**
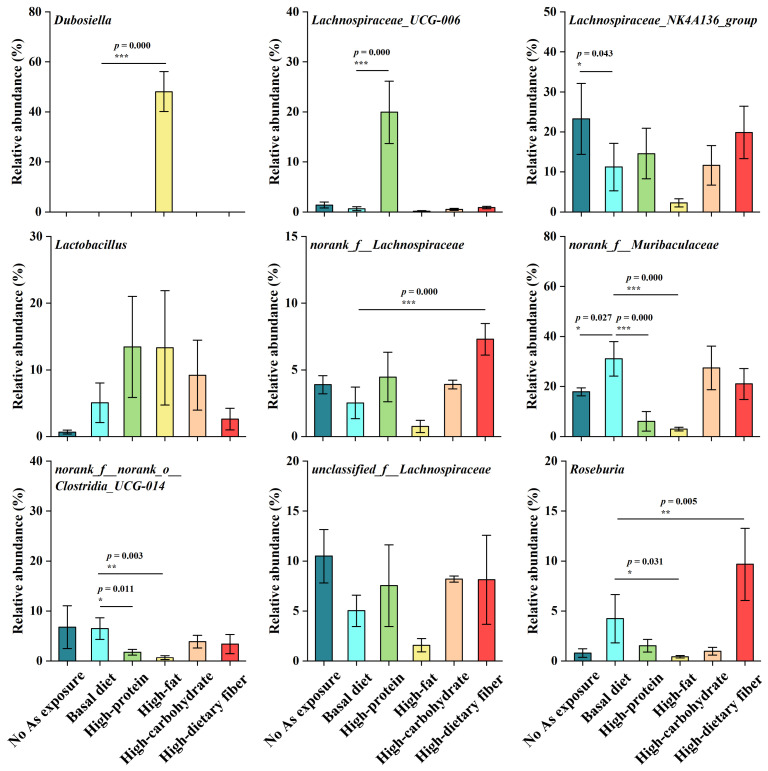
Effects of As exposure and diets on the relative abundance of the top 9 species of the mouse gut microbiota in soil A. No As exposure: mice without As exposure. Marks above the bars indicate significant differences at *p* < 0.05 (*), *p* < 0.01 (**), and *p* < 0.001 (***), respectively.

The results of the genus level showed that As exposure significantly changed the intestinal microbial community structure of mice (Figure 3). Compared with the no-As-exposure group, the relative abundance of *Lachnospiraceae_NK4A136 _ group* (23.3% vs. 11.2%) in the basal diet group was significantly decreased (*p* < 0.05), while the relative abundance of *norank_f__Muribaculaceae* (17.8% vs. 31.1%) was significantly increased (*p* < 0.05). In addition, the relative abundance of *Lactobacillus* (0.68% vs. 5.05%) and *Roseburia* (0.81% vs. 4.24%) showed an upward trend. These changes indicate that the risk of intestinal inflammation is associated with As exposure [46,47].

Dietary structural variations are correlated with shifts in intestinal flora composition under As exposure conditions. High-fat diet significantly increased the relative abundance of *Dubosiella* (0.01% vs. 48.1%, *p* < 0.05) and significantly decreased the relative abundance of *norank_f__Muribaculaceae* (31.1% vs. 3.02%, *p* < 0.05), *norank_f__norank_o__Clostridia_UCG-014* (6.48% vs. 0.68%, *p* < 0.05) and *Roseburia* (4.24% vs. 0.43%, *p* < 0.05). In comparison, the high-protein diet significantly increased the relative abundance of *Lachnospiraceae_UCG-006* (0.66% vs. 19.9%, *p* < 0.05) and significantly decreased the relative abundance of *norank_f__Muribaculaceae* (31.1% vs. 6.06%, *p* < 0.05) and *norank_f__norank_o__Clostridia_UCG-014* (6.48% vs. 1.78%, *p* < 0.05). A high-dietary fiber diet has been reported to exert a positive effect on maintaining the stability of intestinal flora in mice [48,49]. In this study, a high-dietary fiber diet significantly increased the relative abundance of *norank_f__Lachnospiraceae* (2.51% vs. 7.29%, *p* < 0.05) and *Roseburia* (4.24% vs. 9.68%, *p* < 0.05). The relative abundance of *Dubosiella* is up-regulated, which can alleviate intestinal barrier damage caused by inflammatory stimulation [50]. *Roseburia* and *Lachnospiraceae* have been reported to be associated with intestinal barrier protection and anti-inflammatory effects [46,47]. The gut microbiota analysis was based on 16S rRNA gene sequencing, which only reveals compositional correlations. The roles of *Roseburia*, *Lachnospiraceae*, and *Dubosiella* in modulating intestinal barrier function and As absorption remain to be verified by functional experiments. Overall, a high-fat diet increases the risk of As-related intestinal inflammation, while high-protein and high-dietary fiber diets treat inflammation and protect the intestinal barrier by enhancing the relative abundance of probiotics.

### 3.4. Damage of Intestinal Barrier Improved As Absorption

**Figure 4:**
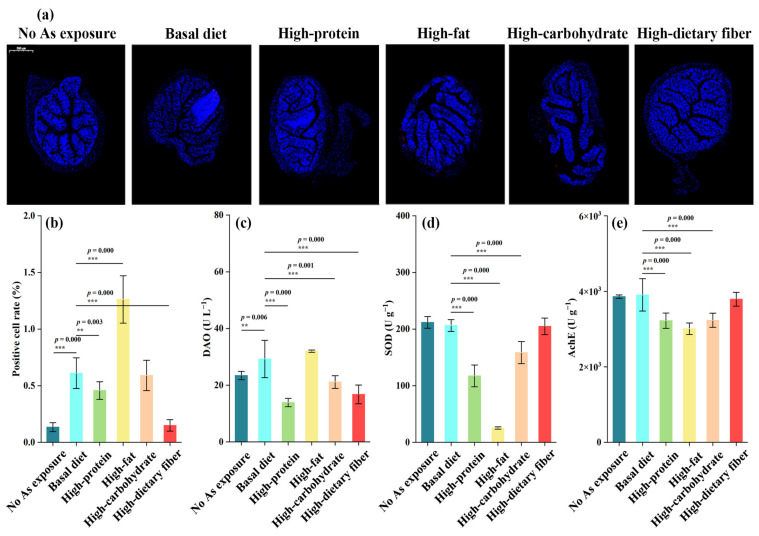
Effects of different dietary structures on small intestinal terminals’ physiological status and intestinal permeability in mice before and after As exposure in soil A. (**a**) TUNEL staining showing the occurrence of apoptosis in the small intestinal terminals of mice caused by exposure to As alone or after supplement of dietary structure. (**b**) Positive cell rate (apoptosis rate) was obtained after counting the number of apoptotic cells via ImageJ software. (**c**) DAO enzyme activity showing changes in intestinal permeability in mice exposed to different diets. Oxidative damage in mice exposed to different dietary groups is shown by SOD (**d**) and AchE (**e**) enzyme activities, respectively. No As exposure: mice fed diet without As exposure. Marks above the bars indicate significant differences at *p* < 0.01 (**), and *p* < 0.001 (***), respectively.

Intestinal epithelial cells serve as the primary physiological barrier against orally ingested arsenic [51]. High-fat diets increase intestinal permeability by reducing the protective probiotics of the intestinal barrier [14]. Positive cell rate was evidenced by TUNEL staining of the ileum tissue sections. Compared to no-As-exposure mice (0.13 ± 0.03%), a significantly increased value was shown in the basal diet mice (0.61 ± 0.09%, *p* < 0.05) (Figure 4a,b). Studies have shown that As exposure increases intestinal epithelial permeability by inducing intestinal inflammation in mice [52].

With the dietary structure strategy adjusted, the positive cell rate was significantly increased in high-fat diet mice (1.26 ± 0.14%, *p* < 0.05) and significantly decreased in high-protein (0.46 ± 0.05%, *p* < 0.05) and high-dietary fiber (0.15 ± 0.03%, *p* < 0.05) diet mice compared to the basal diet mice. The DAO enzyme activity in the high-protein (13.9 ± 0.97 U L^−1^, *p* < 0.05) and high-fiber diet (16.7 ± 2.20 U L^−1^, *p* < 0.05) mice was significantly decreased compared with the basal diet (29.3 ± 4.39 U L^−1^). In conclusion, a high-fat diet may increase intestinal permeability in mice, while high-protein and high-dietary fiber diets improved intestinal permeability affected by As.

Another piece of evidence for apoptosis was the high oxidative damage in the jejunum section of mice fed a high-fat diet. Compared to no-As-exposure mice, As exposure did not cause significant changes in SOD (211 U g^−1^ vs. 206 U g^−1^) and AchE (3860 U g^−1^ vs. 3906 U g^−1^) enzyme activities, suggesting that As-related gut microbiota community changes and inflammation may not be primarily driven by oxidative stress. However, SOD and AchE were significantly decreased in high-protein (117 ± 12.9 U g^−1^ and 3223 ± 135 U g^−1^), high-fat (25.4 ± 1.38 U g^−1^ and 3010 ± 102 U g^−1^), and high-carbohydrate (158 ± 13.1 U g^−1^ and 3234 ± 125 U g^−1^) diets (*p* < 0.05). In contrast, no oxidative damage was found in the jejunum section of mice fed with a high-dietary fiber diet (SOD: 205 ± 9.69 U g^−1^, and AchE: 3791 ± 121 U g^−1^). In general, compared with the basal diet, the high-dietary fiber diet maintained normal SOD and AchE activities and reduced intestinal permeability, which may contribute to lower As absorption.

### 3.5. Mechanisms of Diet Effects on As-RBA

Theoretically, the process of As accumulation in the human body after oral intake includes the following two steps: (1) As in contaminated soil is partially dissolved in the gastrointestinal digestive system and released into the gastrointestinal fluid; (2) dissolved As contacts with intestinal epithelial cells and enters the circulatory system through specific transporters [7].

When the intake of a single food component reached a high level, a significant increase in the stability of the particulate matter was found in the simulated intestinal fluid (Figure S3). The presence of the particulate matter can effectively increase the content of dissolved As in the intestinal phase, which is collectively referred to as As-containing particulate matter. In vitro PBET showed that 1.26–9.89-fold higher As bioaccessibility in the simulated intestinal fluid in the presence of high-fat, high-protein, high-carbohydrate, and high-dietary fiber (Figure 2). However, these results were in contrast to decreased As oral bioavailability in mice with high-protein and high-dietary fiber diets (Figure 2 and Figure S4).

**Figure 5:**
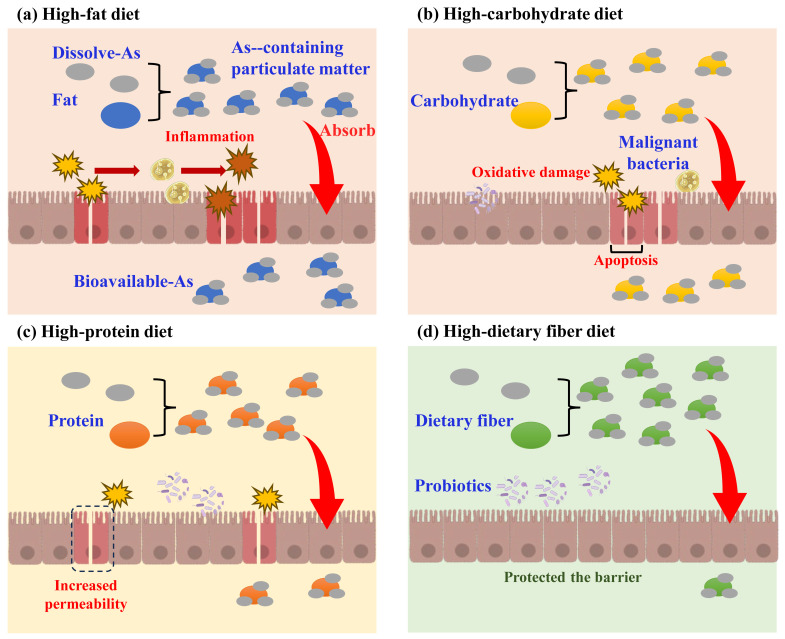
Proposed working models for the regulation of (**a**) high-fat diet, (**b**) high-carbohydrate diet, (**c**) high-protein diet, and (**d**) high-dietary fiber diet in As oral bioavailability.

A high-fat diet led to pronounced decreases in SOD and AchE activities and obvious intestinal barrier impairment, which are associated with oxidative stress and intestinal inflammation. Therefore, a high-fat diet destroyed the intestinal barrier and promoted As-containing particulate matter to pass through the barrier (Figure 5a). High carbohydrates were also correlated with oxidative damage to intestinal tissues but did not significantly affect the relative abundance of intestinal pathogenic bacteria and probiotics. The intestinal permeability of mice on a high-carbohydrate diet was similar to that of mice on a basal diet. Correspondingly, the high-carbohydrate diet showed no significant effect on As-RBA (Figure 2(b2)). Although a high-protein diet was also associated with oxidative damage, it reduced the accumulation of As. Similar studies have reported that protein intake can effectively promote the excretion of As [16]. In this study, the intestinal permeability in high-protein mice was significantly lower than that of the basal diet (Figure 4). This was consistent with the dissolved As forming insoluble complexes with protein in the intestinal phase (pH = 7) [53]. The formation of insoluble complexes inhibited the absorption of As in the intestine. Notably, the intestinal health of mice fed a high-dietary fiber diet was similar to that of mice with no As exposure. The relative abundance of *Roseburia* in the gut microbiota of mice with a high-dietary fiber diet was significantly higher than that of other treatment groups (Figure 3). The butyric acid produced by *Roseburia*, which degrades dietary fiber, can be rapidly absorbed and metabolized by intestinal epithelial cells [54]. Butyric acid can improve the intestinal barrier function by regulating the energy metabolism of intestinal epithelial cells, inhibiting the growth of pathogenic bacteria, and effectively inhibiting the occurrence of intestinal inflammation [49,55,56]. In addition, dietary fiber can effectively promote intestinal peristalsis [32]. Therefore, after arsenic is combined with dietary fiber to form insoluble aggregates, it can accelerate the excretion of As-containing particulate matter and effectively reduce As-RBA (Figure 5d).

### 3.6. Health Risk Assessment of As Exposure Under Different Diets

Health risk assessment was performed using the US EPA standard model based on the following assumptions: (1) oral ingestion is the primary exposure pathway for As; (2) the As-RBA derived from the mouse model is applicable to humans; (3) Monte Carlo simulation minimizes inaccuracies in risk assessment results caused by default parameters.

**Figure 6:**
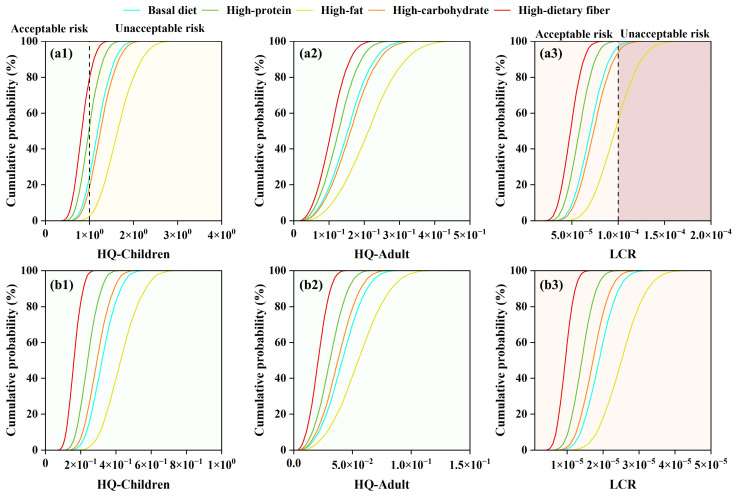
Health risk probability distribution of As under different dietary exposure conditions based on oral bioavailability. (**a1**,**b1**) represent the non-carcinogenic risk hazard quotient (HQ) of children exposed to As in soil A and B, respectively; (**a2**,**b2**) represent the HQ of As in adult exposed soil A and B, respectively; (**a3**,**b3**) represent the lifetime carcinogenic risk (LCR) of As in soil A and B, respectively. The black dash line indicates the unacceptable threshold (10^−4^). The yellow background indicates an acceptable carcinogenic risk, and the red section indicates the lifetime carcinogenic risk.

The As oral bioavailability with different dietary structures was incorporated into the health risk assessment probability model to simulate and predict the effects of different dietary patterns on the health risks of the As-exposed population. This study focused on the oral bioavailability of As, and only the potential health risks of As through oral ingestion were considered (Text S4, Table S4). The results showed that As exposure in both soil A and B resulted in a potential carcinogenic risk exceeding the safety threshold (10^−6^) (Figure 6). Compared with soil B, As exposure in soil A posed unacceptable non-carcinogenic risk (HQ > 1) and lifetime carcinogenic risk (LCR > 10^−4^) to children.

Compared with the basal diet group (1.31, with 97.4% of HQ > 1), high-fat diet increased the probability of non-carcinogenic risk in children exposed to soil A (1.82, with 97.4% of HQ > 1), while high-protein and high-dietary fiber diets effectively mitigated this risk (1.09, with 47.7% of HQ > 1, and 0.91, with 20.7% of HQ > 1, respectively). Notably, a high-dietary fiber diet reduced the average non-carcinogenic risk of children to below the safety threshold, suggesting a potential protective tendency under the model assumptions. Regarding carcinogenic risk, compared with the basal diet group (7.95 × 10^−5^, with 2.9% of LCR > 10^−4^), the high-fat diet resulted in an unacceptable LCR (1.10 × 10^−4^, with 44.0% of LCR > 10^−4^), while the high-dietary fiber diet could control the LCR within an acceptable risk range (5.53 × 10^−5^, with 100% of LCR < 10^−4^). The above results reveal that increasing dietary fiber intake is an effective intervention to reduce the health risk of As for people exposed to As-contaminated soil. However, this health risk assessment provides a screening-level assessment based on mouse As-RBA and default exposure parameters. Due to factors such as individual dietary habits and actual soil intake behavior, the actual risks may be different.

## 4. Conclusions

In this study, the regulation mechanism and health risk effects of different nutrients on the oral bioavailability of soil As were systematically expounded by using in vivo and in vitro methods. Although in vitro experiments have shown that a single high dose of nutrients can promote the dissolution of As in gastrointestinal fluid, the absorption efficiency is closely related to the intestinal barrier function. A high-fat diet significantly promoted As absorption and increased the health risk by 1.33–1.38-fold by inducing oxidative stress injury, leading to intestinal microbial inflammation and destroying intestinal permeability. On the contrary, a high-dietary fiber diet effectively inhibited As absorption by inhibiting oxidative stress, promoting the relative abundance of intestinal probiotics and the integrity of the intestinal barrier, and reducing the risk to 0.50–0.70-fold of the basal diet group.

The present study focused on short-term exposure in mice, and the markers of oxidative stress and intestinal damage were measured at a single endpoint. The changes reflected the combined effects of As + diet exposure, rather than being caused by the diet itself. In addition, although a high-protein diet inhibits As absorption, it also induces oxidative damage, which may increase the risk of long-term inflammation. In the future, it is necessary to carry out long-term trials to evaluate the health effects of dietary interventions on As-exposed populations. Only wheat fiber was used in the present study. Further studies using various fiber sources are needed to compare their effects on arsenic bioavailability. Overall, this study unveils the underlying mechanisms by which dietary fiber and fat regulate As oral bioavailability and cumulative health risks via modulating intestinal barrier integrity. These results provide a theoretical basis for further research on the use of dietary fiber to improve intestinal barrier health and reduce As absorption, aiming to reduce arsenic-related health risks in contaminated areas through targeted dietary strategies.

## Data Availability

The original contributions presented in the study are included in the article/Supplementary Materials, and further inquiries can be directed to the corresponding authors.

## Supplementary Materials

The following supporting information can be downloaded at: https://www.mdpi.com/article/10.3390/foods15111961/s1, Text S1: As bioaccessibility via PBET method; Text S2: TUNEL staining of intestinal tissue; Text S3: Determination of oxidative damage in the intestine; Text S4: Health risk assessment; Figure S1: Linear relationship between As concentration in mouse liver and kidneys dosed as sodium arsenate; Figure S2: The changes in body weight and average daily dietary consumption of mice during different dietary exposures; Figure S3: Zeta potential of simulated intestinal fluid under different diets; Figure S4: As bioavailability and bioaccessibility of intestinal phase were compared for (a) soil A and (b) soil B. Different letters on the columns indicate significant differences between As bioavailability and bioaccessibility (*p* < 0.05); Figure S5: Effects of As exposure and different dietary supplements on the α diversity of intestinal microbial community in mice; Figure S6: The effects of arsenic exposure and different dietary patterns on the intestinal microbial community structure of mice were analyzed by β diversity. Non-metric multidimensional scaling (NMDS) based on Bray–Curtis distance revealed the differences in the community composition of intestinal microorganisms at the OTU level in each treatment group; Figure S7: Effects of different diets on the structure of the gut microbiota in mice. The distribution of gut microbiota in mice under different diets was analyzed at the phylum level and represented using Circos plots (a). The ratio of Firmicutes to Bacteroidetes (F/B) in gut microbes was used as an indicator of whether the gut microbiota was dysbiosis (b); Figure S8: Differences in gut microbiota of mice on different diets. (a) Composition of gut microbes at the genus level in mice CK mouse chew; (b) composition of gut microbes at the genus level in mice under As exposure; and (c–f) represent the composition of gut microbes in mice under As exposure with high protein, high fat, high carbohydrate, and high-dietary fiber diet, respectively; Table S1: Physical and chemical properties of two kinds of contaminated soil; Table S2: Nutrient distribution composition in different dietary amendments; Table S3: Composition of the diet prepared by different food and mouse chow mixing with contaminated soil and determine the concentration of arsenic in the diet to calculate the relative bioavailability of As under the exposed to diet with As; Table S4: As exposure health risk assessment model calculated parameters and values. References [57,58,59,60,61] are cited in the Supplementary Materials.
