# Supplementary material for: High-Dietary Fiber Diet Reduces Arsenic Oral Bioavailability and Health Risk from Soils by Regulating Gut Microbiota and Intestinal Barrier Function
Source: Foods. 2026 Jun 2;15(11):1961. doi: 10.3390/foods15111961 (PMC13257037; doi:10.3390/foods15111961)
Supplement: Supplementary file 1 [file foods-15-01961-s001.zip › foods-4313555-supplementary.pdf]

High-dietary fiber diet reduces arsenic oral bioavailability and health risk from soils by regulating gut microbiota and intestinal barrier function

Shuo Chen[1], Lei Han[2], Enfeng Liu[1], Hongbo Li[3*], Jie Li[1*]

[1] Shandong Province Key Laboratory of Emerging Contaminants Risk Prevention and Control, College of Geography and Environment, Shandong Normal University, Jinan 250358, China

[2]Shandong Provincial Geo-mineral Engineering Exploration Institute, Shandong Provincial Bureau of Geology & Mineral Resources, Jinan, 250014, China

[3]State Key Laboratory of Pollution Control and Resource Reuse, Jiangsu Key Laboratory of Vehicle Emissions Control, School of Environment, Nanjing University, Nanjing 210023, China

[*] Correspondence: Hongbo Li, @nju.edu.cn; Jie Li, lijie2017@sdnu.edu.cn.

Text 1: As bioaccessibility via PBET method

The configured simulated gastric fluid (pH 2.50) contained 1.25 g L^{-1} pepsin, 0.5 g L^{-1} sodium malate, 0.5 g L^{-1} sodium citrate, 0.42 mL L^{-1} lactic acid and 0.5 mL L^{-1} acetic acid. 20 mL of simulated gastric fluid was extracted with a mixture containing 0.2 g of contaminated soil + 1 g of dietary ingredient supplements for 1 h at 37 °C, and then the simulated fluid pH was adjusted to 7.00, and the intestinal-phase simulated fluid was obtained by supplementing with bile (1.75 g L^{-1}) and pancreatin (0.5 g L^{-1}). The simulated intestinal fluid was extracted continuously for 4 h. The supernatant was centrifuged and filtered through 0.45 μm filter membrane (MCE) and analyzed for As content using ICP-Ms. As bioaccessibility was calculated by dividing the extractable As by the total As in soil. For QA/QC, SRM NIST 2711a was included, with bioaccessible As in IP was 48.3 ± 0.23 mg kg-1, consistent with 49.3 ± 2.94 mg kg-1 reported by Li et al. (2015) [57].

Text S2: TUNEL staining of intestinal tissue

1 Deparaffinize and rehydrate: Put the slices in 3 changes of biodewax and clear solution, 10min each, then dehydrate in 3 changes of pure ethanol for 5min each, wash in distilled water.

2 Protease K repair: After the sections were slightly dried, a circle was drawn around the tissue with a histochemical pen (to prevent the liquid from flowing away), and proteinase K working solution was dropped into the circle to cover the tissue, and the tissue was incubated at 37℃ for 20min. The slides were washed 3 times in PBS (PH7.4) by shaking on a decolorization shaker, 5min each time. (Preparation method of proteinase K working solution, stock solution: PBS=1:9)

3 Permeabilization (optional) : After the sections were slightly dried, the membrane breaking solution was added in the ring to cover the tissue, and the slides were incubated at room temperature for 20min. The slides were placed in PBS (PH7.4) and washed 3 times, 5min each time, by shaking on a decolorizing shaker.

4 Equilibrium at room temperature: After slightly drying the slides, the tissues were covered by dropping buffer in the ring and incubated with buffer at room temperature for 10min.

5 Adding reaction solution: Appropriate amount of TDT enzyme, dUTP and buffer in tunel kit were mixed according to the number of slices and tissue size at a ratio of 2:5:50, and added to the circle to cover the tissue. The sections were placed flat in a wet box, incubated at 37℃ for 1 hour, and a small amount of water was added to the wet box to maintain humidity.

6 DAPI counterstain in nucleus: Wash three times with PBS (pH 7.4) in a decoloring shaker, 5min minutes each time. After removing PBS, DAPI solution was dripped into the circle and incubated at room temperature for 10min in the dark.

7 Mount: The slides were washed 3 times with PBS (PH7.4) for 5min each time. After the slide was slightly dried, the cell side down was sealed on the slide with anti-fluorescence quenching sealing agent.

8 Image acquisition: The sections were observed under a fluorescence microscope and images were collected. (DAPI UV excitation wavelength was 330-380nm, emission wavelength was 420nm, emitting blue light; TMR has an excitation wavelength of 510-561nm and an emission wavelength of 590 nm, emitting red light.)

Text S3: Determination of oxidative damage in the intestine

Fresh jejunum samples (~0.1 g) were ground into a pulp at 4°C, and the extent of oxidative damage in mice after As + different diets exposure was quantified using Acetylcholinesterase (AchE) Assay Kit (BC2020, Beijing Solarbio Science & Technology Co., Ltd.) and Superoxide Dismutase (SOD) Assay Kit (BC0170, Beijing Solarbio Science & Technology Co., Ltd.), respectively. Six replicates were set up for each of all treatment groups. All experimental manipulations were performed in strict accordance with the instructions provided with the corresponding enzyme assay kits.

Text S4: Health risk assessment

According to the recommendations of the US EPA (2018), the health risk assessment method divides the potential health risks of As into non-carcinogenic risk (NCR) and lifetime carcinogenic risk (LCR) [58]. To accurately assess the potential health risks of As exposure to children in different dietary situations, this study incorporated the oral bioavailability of As under different dietary treatments into the health risk assessment method. According to physiological variations, the simulated population was divided into children and adults, and then the average daily dose (ADD_{ingest}, mg kg^{-1} day^{-1}) was used to predict the exposure through the ingestion pathway. The non-carcinogenic risk of adults and children was calculated by the quotient of ADD_{ingest} and As reference dose (*RfD*). The lifetime carcinogenic risk was calculated by multiplying the lifetime average daily dose (*LADD*) with the slope factor (SF_i) of As to assess an individual exposure to carcinogenic risk during a lifetime with different dietary habits. The calculation formulas are as follows:

$$ADD_{ingest} = (C_i \times R_{ingest} \times EF \times ED \times 10^{-6})/(BW \times AT),$$

$$HQ = ADD_i/RfD_i,$$

$$LADD_{ingest} =$$

$$C_i \times EF \times (R_{ingest} \times ED_{Child}/BW_{Child} + R_{ingest} \times ED_{Adult}/BW_{Adult}) \times 10^{-6}/AT,$$

$$LCR = LADD_i \times SF_i,$$

where C_i represents the bioavailable-As content (mg kg^{-1}), R_{ingest} represents the ingestion rate (kg d^{-1}), *EF* is the exposure frequency (d year^{-1}), *ED* represents the exposure duration (year), *BW* denotes human body weight (kg), and *AT* represents the period over which the dose was averaged (d). The potential non-carcinogenic effect could occur when HI > 1, while if TLCR > 10^{-4}, it indicated a significant lifetime carcinogenic risk. The acceptable level of carcinogenic risk was when TLCR < 10^{-6}.

In addition, the Monte Carlo simulation method was employed for health risk assessment to avoid overestimating or underestimating risks due to the use of deterministic parameters [59, 60]. Uncertainty mainly arises from parameter variability, cross-species extrapolation, and single-point bioavailability measurement. Monte Carlo simulation provided cumulative probability distributions to quantify uncertainty in HQ and LCR. In Crystal Ball 11.1.2.4, 10000 Monte Carlo simulations of the frequency and cumulative probability of health risks for population exposed to PTEs were performed [61].

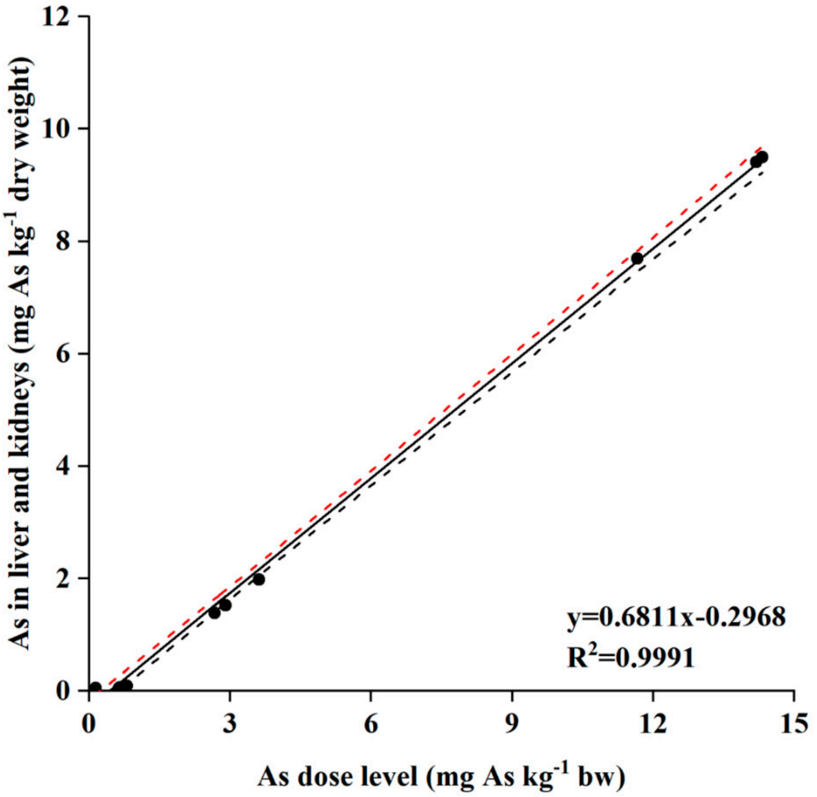

Figure S1 Linear relationship between As concentration in mouse liver and kidneys dosed as sodium arsenate.

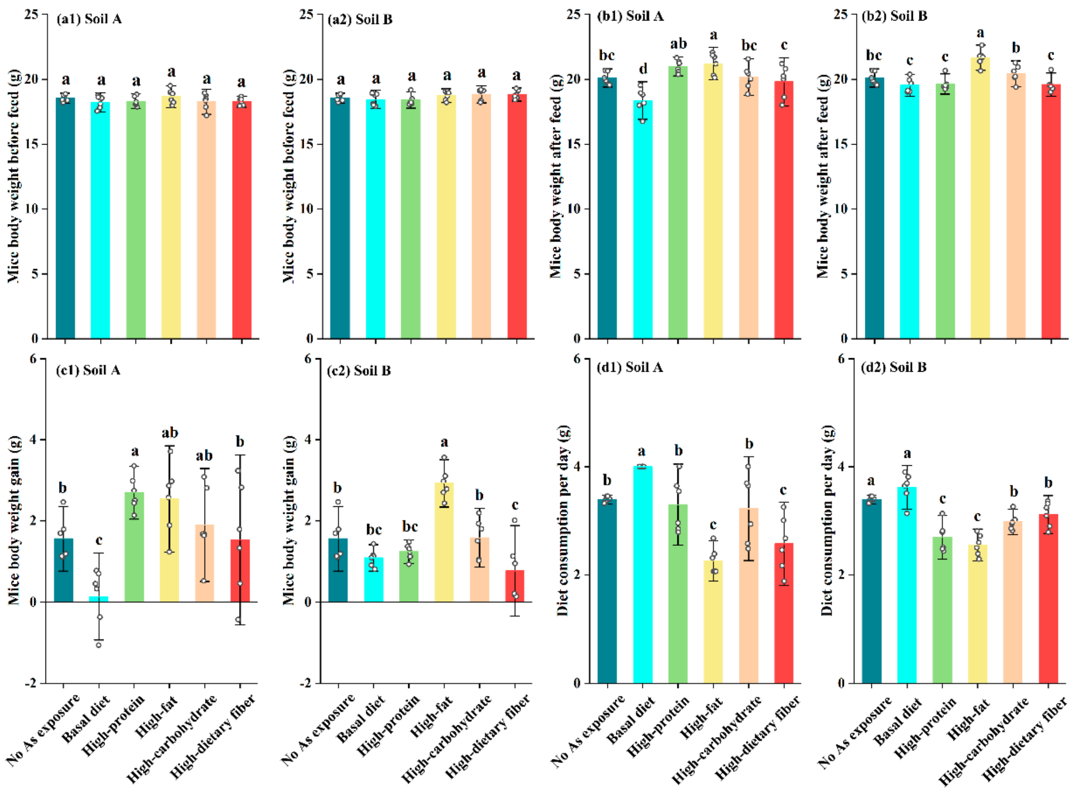

Figure S2 The changes in body weight and average daily dietary consumption of mice during different dietary exposures.

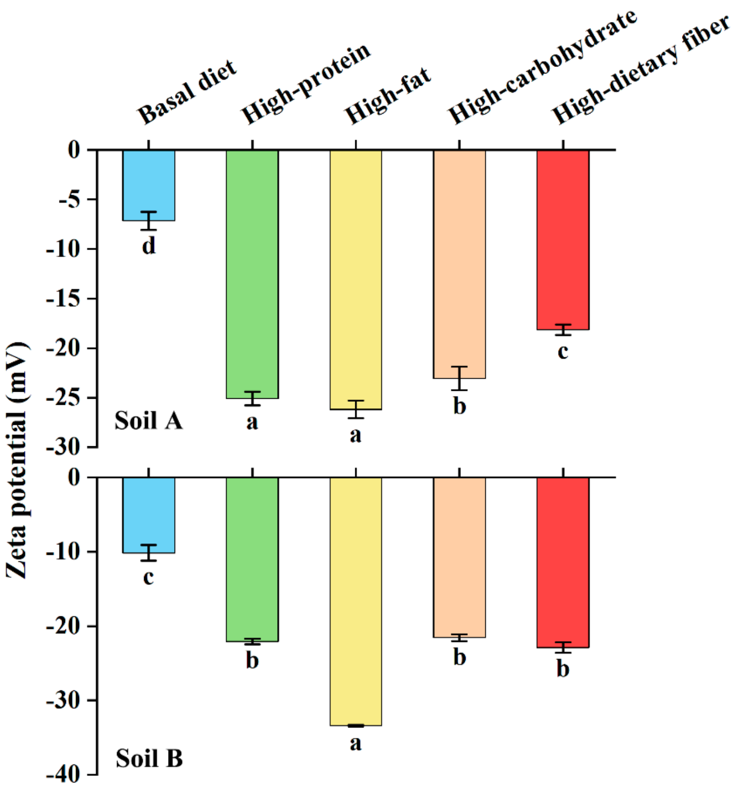

Figure S3 Zeta potential of simulated intestinal fluid under different diets.

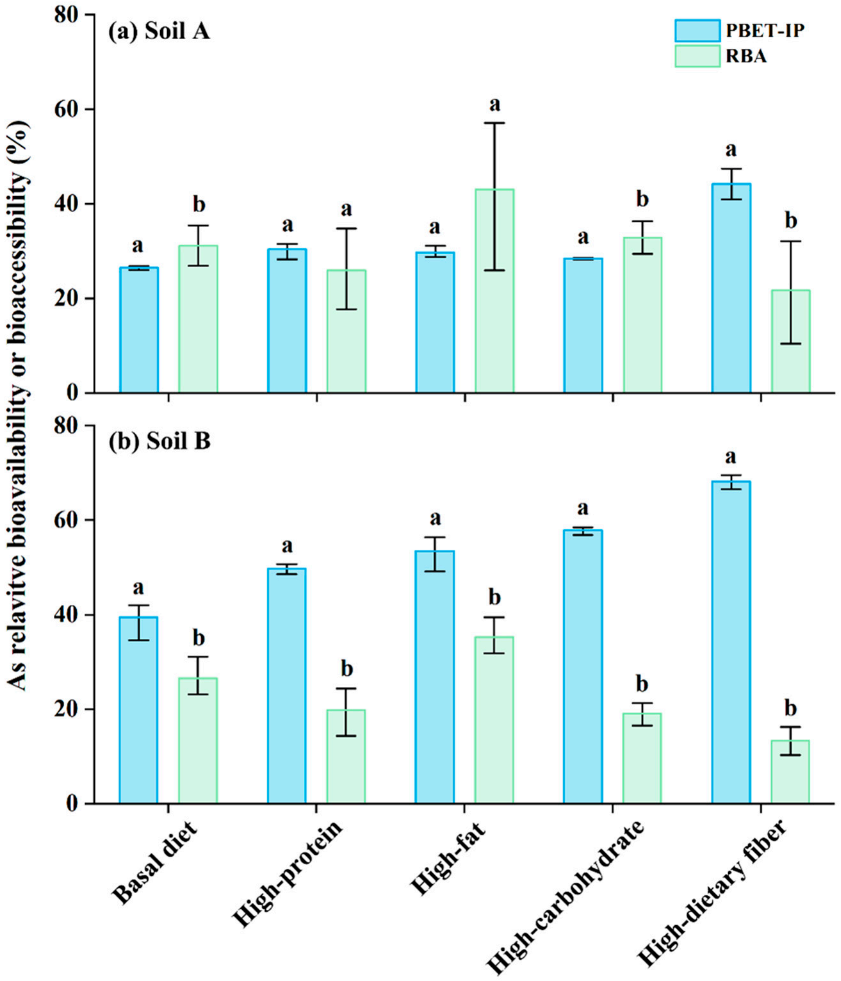

Figure S4 As bioavailability and bioaccessibility of intestinal phase were compared for (a) soil A and (b) soil B. Different letters on the columns indicate significant differences between As bioavailability and bioaccessibility ($p < 0.05$).

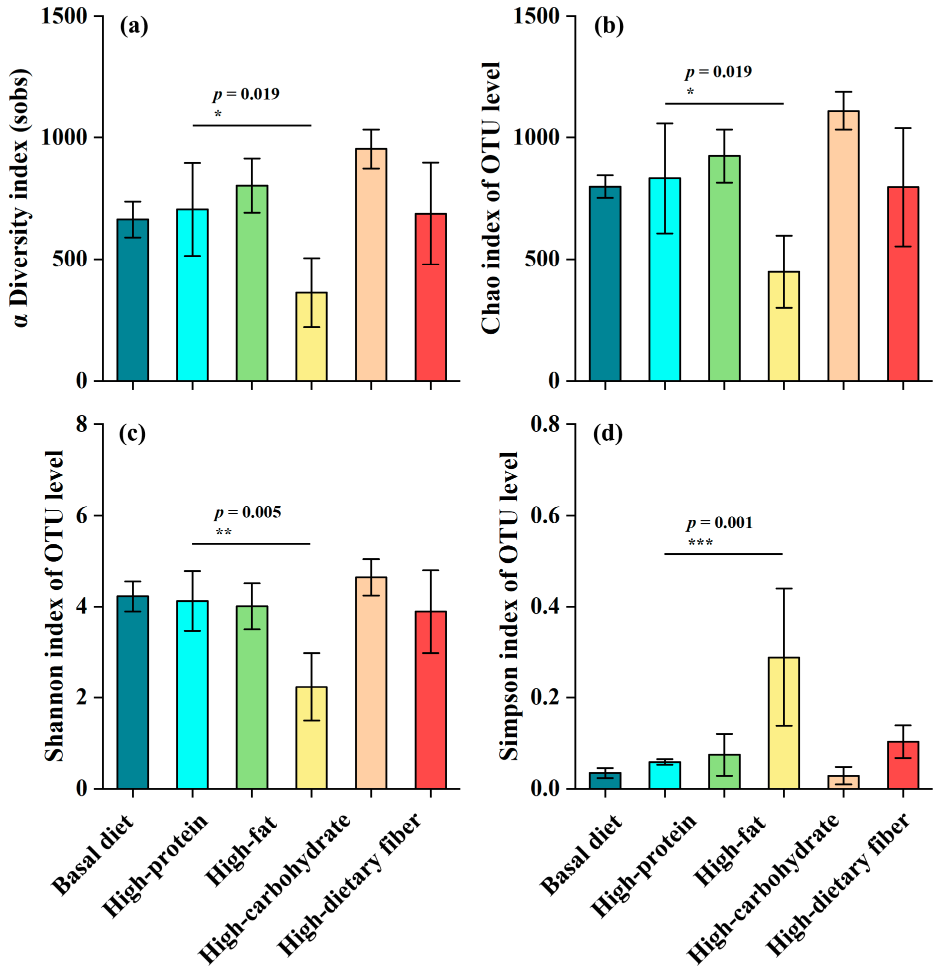

Figure S5 Effects of As exposure and different dietary supplements on the α diversity of intestinal microbial community in mice.

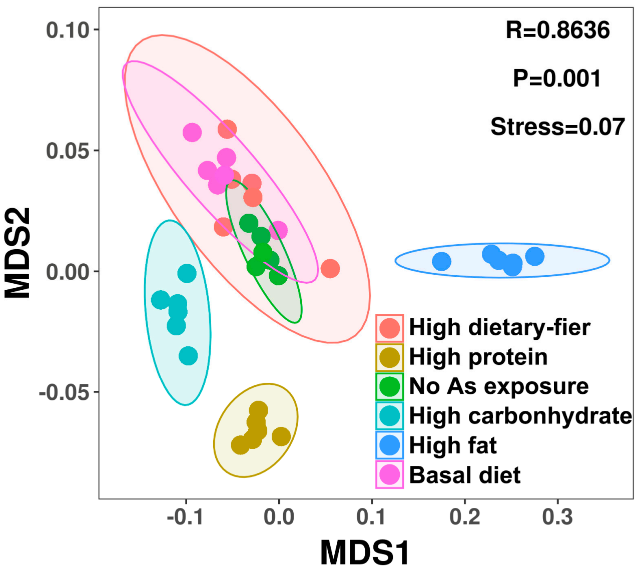

Figure S6 The effects of arsenic exposure and different dietary patterns on the intestinal microbial community structure of mice were analyzed by β diversity. Non-metric multidimensional scaling (NMDS) based on Bray-Curtis distance revealed the differences in the community composition of intestinal microorganisms at the OTU level in each treatment group.

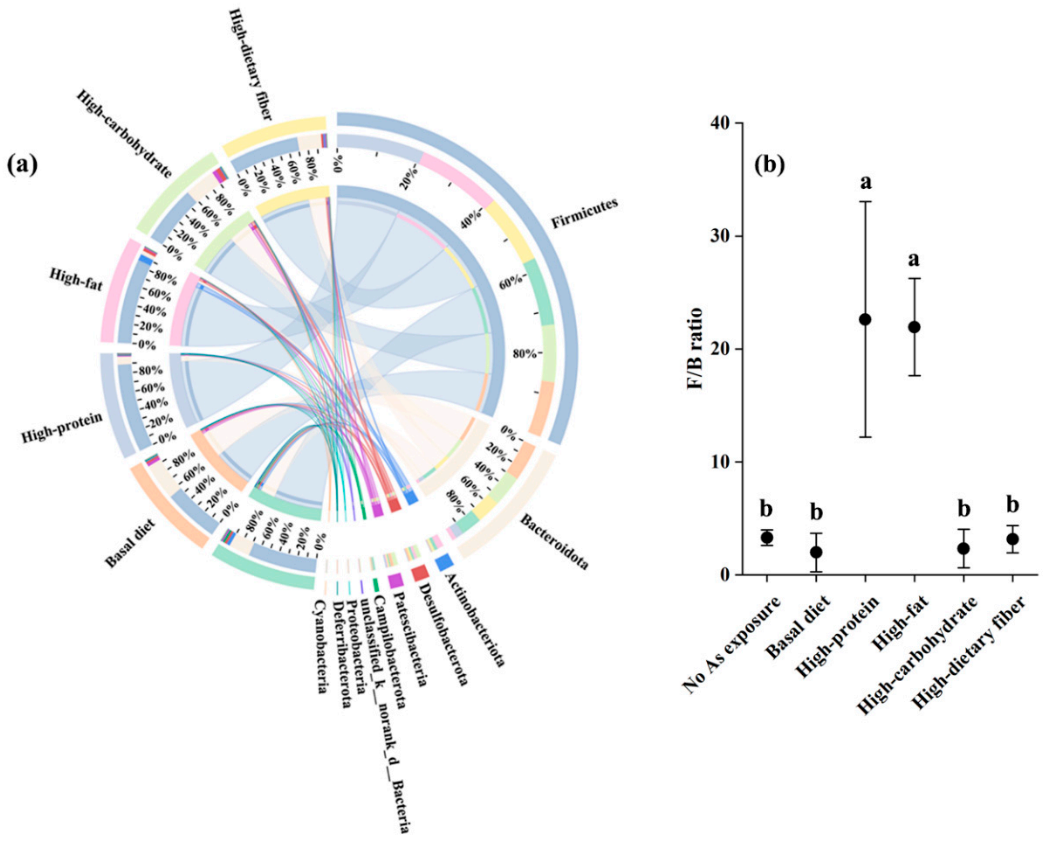

Figure S7 Effects of different diets on the structure of the gut microbiota in mice. The distribution of gut microbiota in mice under different diets was analyzed at the phylum level and represented using Circos plots (a). The ratio of *Firmicutes* to *Bacteroidetes* (F/B) in gut microbes was used as an indicator of whether the gut microbiota was dysbiosis (b).

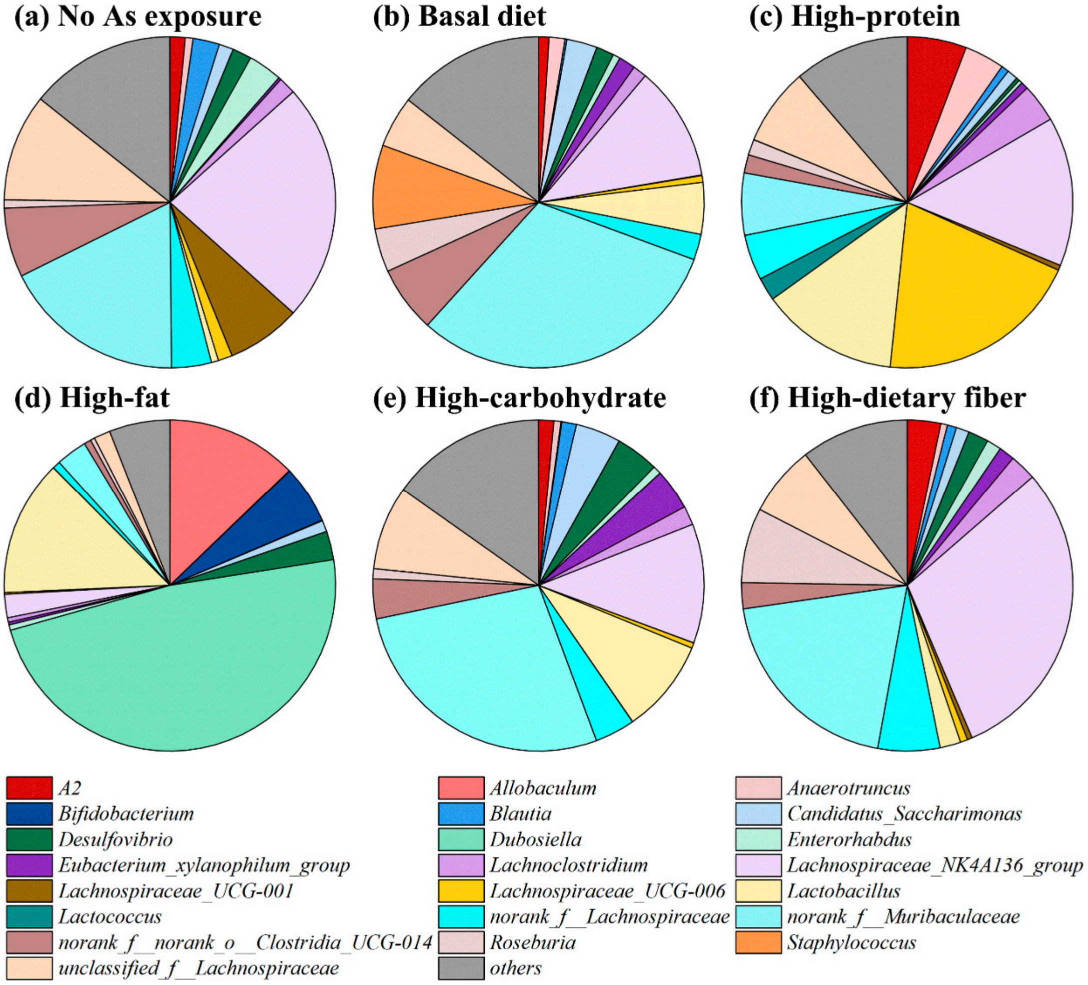

Figure S8 Differences in gut microbiota of mice on different diets. (a) Composition of gut microbes at the genus level in mice CK mouse chew; (b) composition of gut microbes at the genus level in mice under As exposure; and (c)-(f) represent the composition of gut microbes in mice under As exposure with high protein, high fat, high carbohydrate, and high dietary fiber diet, respectively.

Table S1 Physical and chemical properties of two kinds of contaminated soil

	pH	OM (%)	Fe (g kg^{-1})	Ca (g kg^{-1})	Mn (g kg^{-1})	As (mg kg^{-1})	P (g kg^{-1})
Soil A	5.02	8.33	89.1±9.53	10.6±0.02	1.91±0.11	381±7.71	0.56±0.02
Soil B	8.02	4.98	22.4±1.68	14.8±0.21	0.49±0.04	121±9.58	0.29±0.01

Table S2 Nutrient distribution composition in different dietary amendments.

	Carbohydrate (g/100g)	Protein (g/100g)	Fat (g/100g)	Fiber (g/100g)	Energy (KJ/100g)
Basal diet	51.8	21.9	5.03	4.25	1474
High-protein	40.2	37.6	4.45	3.29	1513
High-fat	43.7	18.5	19.3	3.59	1800
High-carbohydrate	67.4	14.8	2.90	3.19	1531
High-dietary fiber	42.5	20.0	4.62	16.7	1367

The carbohydrate, protein, fat and fiber content were determined using the Chinese national food safety standard methods GB28050-2011, GB5003.5-2016, GB5009.6-2016 and GB5009.88-2014. The energy is calculated according to GB28050-2011 standard, and the formula is as follows: Energy (kJ/100 g) = 37 kJ/g × fat content (g/100 g) + 17 kJ/g × (protein content (g/100 g) +17 kJ/g × carbohydrate content (g/100 g) + 8 kJ/g × fiber content (g/100 g).

Table S3 Composition of the diet prepared by different food and mouse chow mixing with contaminated soil and determine the concentration of arsenic in the diet to calculate the relative bioavailability of As under the exposed to diet with As.

	Food/g	Mouse chow/g	Soil/g	As in prepared diet (mg kg^{-1})	
				Soil A	Soil B
Basal diet	-	348.00	12	12.7	4.04
High-protein	174	174	12	12.7	4.04
High-fat	54	294	12	12.7	4.04
High-carbohydrate	174	174	12	12.7	4.04
High-dietary fiber	104.4	243.6	12	12.7	4.04

Table S4 As exposure health risk assessment model calculated parameters and values.

Parameter	Children	Adult
EF (day $year^{-1}$)	TRI (180, 345, 365)	
R_{ingest} (mg day^{-1})	TRI (66, 103, 161)	TRI (4, 30, 52)
ED (years)	6	24
BW (kg)	29.3	60
AT (day)	365 × ED (non-carcinogenic)<br>365 × 70 (carcinogenic)	
RfD(mg $(kg·d)^{-1}$)	3.00×10^{-4}	
SF((kg·d) mg^{-1})	1.50	
